# Chloral in Toothache

**Published:** 1872-11

**Authors:** 


*Chloral in Toothache.*—A correspondent of the *Br. Med. Jour.* writes that, for some time, he has "been in the habit of using chloral hydrate, not only as an internal sedative in cases of severe dental neuralgia and of caries where it was at the time inadvisable to permit extraction, but more frequently as a direct local application to the carious tooth. A few grains of the solid hydrate introduced into the cavity of the tooth with a quill, speedily dissolve there; and in the course of a few minutes, during which a not unpleasant warm sensation is experienced, the pain is either deadened, or more often effectually allayed. A second or third application may be resorted to if necessary." In the last instance under his notice, "a young lady in a delicate state of health, suffering intensely from a carious tooth, received immediate relief from this application. The toothache was relieved in a few minutes; aching did not return all night; and when it did in the morning, was again stopped by the same means."

